# Technostress Creators and Outcomes Among Egyptian Medical Staff and Students: A Multicenter Cross-Sectional Study of Remote Working Environment During COVID-19 Pandemic

**DOI:** 10.3389/fpubh.2022.796321

**Published:** 2022-04-26

**Authors:** Zeinab A. Kasemy, Asmaa F. Sharif, Ayah M. Barakat, Shaimaa R. Abdelmohsen, Nancy H. Hassan, Nagwa N. Hegazy, Asmaa Y. Sharfeldin, Angham S. El-Ma'doul, Kholoud Adel Alsawy, Hanaa M. Abo Shereda, Sally Abdelwanees

**Affiliations:** ^1^Department of Public Health and Community Medicine, Faculty of Medicine, Menoufia University, Shibin El Kom, Egypt; ^2^Department of Forensic Medicine and Clinical Toxicology, Faculty of Medicine, Tanta University, Tanta, Egypt; ^3^Department of Clinical Medical Sciences, College of Medicine, Dar Al Uloom University, Riyadh, Saudi Arabia; ^4^Department of Family Medicine, Faculty of Medicine, Menoufia University, Shibin El Kom, Egypt; ^5^Department of Anatomy and Embryology, Faculty of Medicine for Girls, Al-Azhar University, Cairo, Egypt; ^6^Department of Human Anatomy and Embryology, Faculty of Medicine, Zagazig University, Zagazig, Egypt; ^7^Department of Psychiatric and Mental Health Nursing, Faculty of Nursing, Menoufia University, Shibin El Kom, Egypt

**Keywords:** teleworking, work engagement, medical staff, COVID-19, coenzyme Q10, Egypt, burnout, technostress

## Abstract

**Objectives:**

This study aimed to investigate the technostress creators and outcomes among University medical and nursing faculties and students as direct effects of the remote working environment during the COVID-19 pandemic.

**Background:**

Due to the current COVID-19 pandemic, shifting to virtual learning that implies utilizing the information and communication technologies (ICTs) is urgent. Technostress is a problem commonly arising in the virtual working environments and it occurs due to misfitting and maladaptation between the individual and the changeable requirements of ICTs.

**Methods:**

A multicenter cross-sectional study was conducted in medicine and nursing colleges of 5 Egyptian universities and included both staff members and students. The data were collected through personal interviews, from January to May 2021. All the participants took a four-part questionnaire that asked about personal and demographic data, technostress creators, job or study, and technical characteristics and technostress outcomes (burnout, strain, and work engagement). Furthermore, participants' blood cortisol and co-enzyme Q10 (CoQ10) levels were tested in a random sample of the students and medical staff.

**Results:**

A total of 3,582 respondents participated in the study, 1,056 staff members and 2,526 students where 33.3% of the staff members and 7.6% of students reported high technostress. Among staff members, total technostress score significantly predicted Cortisol level (β = 2.98, CI 95%: 0.13-5.83), CoQ10(β = −6.54, CI 95%: [(−8.52)–(−4.56), strain (β = 1.20, CI 95%: 0.93–1.47), burnout (β = 0.73, CI 95%: 0.48–0.97) and engagement (β = −0.44, CI 95%: [(−0.77)–(−0.11)]) whereas among students, total technostress score significantly predicted cortisol level (β = 6.64, CI 95%: 2.78–10.49), strain (β = 1.25, CI 95%: 0.72–1.77), and burnout (β = 0.70, CI 95%: 0.37–1.04). Among staff members and students, technology characteristics were significantly positive predictors to technostress while job characteristics were significantly negative predictors to technostress.

**Conclusion:**

The Egyptian medical staff members and students reported moderate-to-high technostress which was associated with high burnout, strain, and cortisol level; moreover, high technostress was associated with low-work engagement and low CoQ10 enzyme. This study highlighted the need to establish psychological support programs for staff members and students during the COVID-19 pandemic.

## Introduction

The COVID-19 pandemic has forced millions of people around the world to adopt a remote work environment using a variety of online platforms. Previous research reported that due to the current pandemic, nearly 50% of the employees were working from home compared with 12% before the pandemic (1).

Among the sectors that have been negatively affected by this transformation are health care, in particular, the capabilities of staff and academics (2).

In general, virtual work negatively affected human emotional and behavioral characteristics. For example, reported adverse effects include social isolation, breakdown of social relationships, and increased family and work conflicts (3–5).

The transition to a virtual work and learning environment requires the adoption of information and communication technologies. However, despite the potential advantages and benefits of using ICTs in higher education, the capabilities of human beings to keep pace with the rapid changes in ICTs are still limited. Among other populations, healthcare workers and medical students are highly exposed to what is known as technical stress which is the stress they are exposed to as a result of their inability to adapt to the changing demands of technology (6, 7).

Stress is the negative feeling of vulnerability due to environmental requirements exceeding existing resources. Given this, technical stress is the negative feeling resulting from the inability of individuals to handle advanced technology to meet business demands (8, 9).

Technostress has been a common feature among people who work in virtual environments. Researchers have documented the negative effects of technology on human health. Aside from the behavioral strain caused by constant exposure to ICT, documented negative effects of technostress include frequent eye strain, headaches, high blood pressure, back pain, stomach problems, irritability, and heart attacks (10). It has been found that these ill-effects reduce employee quality of performance, job satisfaction, and ongoing commitment (7).

Although previous researchers have recognized in different learning contexts and sought to express technological stressors, the scientists have not provided any empirical findings about the links between technostress and workplace outcomes (11–13).

### The Aim of the Work

Through current research, we contribute to the current literature by extending estimates of the prevalence of technostress, determining its consequences in unpredictable work environments, and highlighting negative emotional responses to technology. Additional objectives of this study were to provide a model for predicting the continued use of online platforms among healthcare staff and medical students and to assess the relationships between emotional and motivational aspects (burnout, strain, and work engagement) and continued use of ICTs. Adding to the previous literature, we have investigated the harmful effects of technology on human biological systems.

## Methods

### Study Design and Setting

A cross-sectional, multicenter study was conducted from January to May 2021 on 3,582 participants recruited from five randomly selected medical and nursing schools.

### Sampling and Sample Size

Since the prevalence of technostress was not known among the students or the staff, and the occurrence and non-occurrence were equal to 0.50, the sample size at 95% CI was calculated using the following equation: *n* = Z^2^P(1–P)/d^2^ where *n* represents the sample size, *Z* is the confidence level, and *P* stands for the expected proportion. For staff members, the sample size was estimated at 1,114 participants. The expected dropout rate was 10%, and 1,127 questionnaires were distributed. We received 1,056 completed questionnaires, with a response rate of 93.7%. The student sample size was estimated at 2,144; therefore, we distributed 2,600 surveys based on an expected dropout rate of 15%. We received 2,526 completed questionnaires from students, which is an astounding response rate of 97.2%.

Among the five randomly selected medical schools, a proportional allocation method was applied to determine the appropriate number of participants, so all the students and staff members in these five schools of medicine and nursing were assigned. Each contributing school has an academic and clinical department with different academic degrees. Accordingly, staff members were selected proportionately according to department and academic degree. In parallel, participating students were selected in the same way in proportion to the college and year.

### Data Collection

Between educational sessions and during break times, students and staff were interviewed at randomly selected schools. Rest time was defined as the time off from work or study before work or during the day. It should not be at the end of the day. It was chosen to encourage participants to participate freely without creating an undue burden. The data were collected by a qualified team consisting of dedicated students, staff, and nurses from each college and accustomed to the schedule of staff and students in their school. This team underwent a two-day training workshop on applied questionnaires followed by testing to avoid inter-observer and intra-observer bias. A nurse was attending with the team to draw the blood sample. Members of the trained team interviewed staff and students from five different colleges of medicine and nursing and then left questionnaires for staff to complete; for the students, the trained team collected data on the spot using an interview-based questionnaire.

### Inclusion and Exclusion Criteria

Being an Egyptian staff member or student affiliated with the college of Medicine or Nursing was the main inclusion criteria. Additional criteria were added for staff members including working as regular full-time employed, according to their contract with the University, with no minimum years of experience. For students, they should be regularly registered for the previous levels without withdrawal or postponing any previous level.

Exclusion criteria were being non-Egyptian, affiliated with other affiliations, previous withdrawal or postponing level(s) for students, and working as a part timer or adjunct staff.

### Data Collection Tool

A pilot study was conducted with 20 University staff members and 25 students to validate the questionnaire. Participants in the pilot study were excluded from the study. Public health and family medicine professionals evaluated the questionnaire for its suitability, ability to properly measure technostress, and its effects on work and study outcomes. Cronbach's alpha calculated for the study survey was 0.81, indicating good reliability.

**Figure 1:**
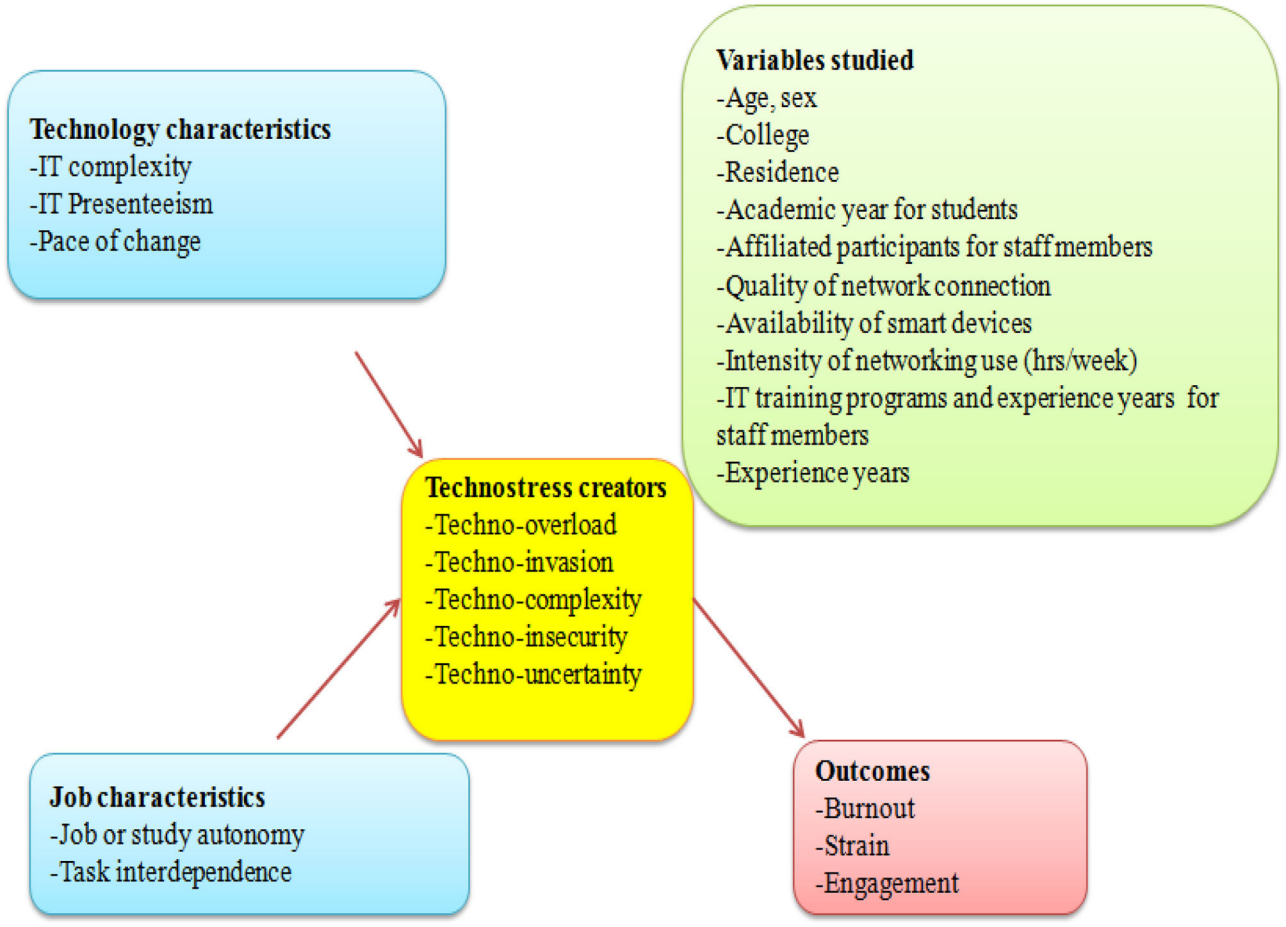
Conceptual theoretical framework of the current study.

Figure 1 presents the theoretical and conceptual framework for this study. All the participants underwent the following:

#### Completing a Predesigned Questionnaire Composed of Four Parts

Personal and demographic data (nine questions on student questionnaire, 11 on staff questionnaire): This questionnaire included age, gender, college, University, residence (urban and rural), perceived quality of network connection, availability of smart devices, and an average number of hours spent using ICT, in addition to the academic year for student participants. Staff participants were asked their affiliated department (academic or clinical), whether they had attended any ICT training programs and their years of experience.

#### Technostress (23 Questions)

To measure technostress, we used the scale developed by Ragu-Nathan et al. (12**)**, which measures the phenomenon on five dimensions: techno-overload (five questions), techno-invasion (four questions), techno-complexity (five questions), techno-insecurity (five questions), and techno-uncertainty (four questions) (14). Respondents rate the items on 5-point Likert scales that range from 1 (strongly disagree) to 5 (strongly agree), and higher total scores indicate greater technostress (15).

#### Job or Study and Technology Characteristics (16 Questions)

Job or study autonomy (three questions), task interdependence (three questions), IT complexity (three questions), IT presenteeism (four questions), and pace of IT change (three questions) (16).

#### Technostress Outcomes (21 Questions)

We measured strain (three questions) by asking participants about feeling drained from activities that require using IT, feeling tired from IT activities, and whether working all day with IT is a strain. We measured burnout using (17) burnout questionnaires (nine questions), and we measured engagement with the Utrecht Work Engagement Scale (nine questions) (18). Participants rated all technostress outcomes on 5-point Likert scales that ranged from 1 (strongly disagree) to 5 (strongly agree).

#### Blood Sampling

A team of nurses collected the blood sample under sterile conditions. AM samples were collected from 151 staff members and 122 students who were randomly selected from those who answered the questionnaires and then the sample was categorized according to standard protocols. We measured serum cortisol using an electrophoretic immunoassay in a Cobas e601 automatic analyzer (Roche Diagnostics, Mannheim, Germany). The collected samples were gently inverted 5 times immediately after withdrawal without shaking, allowing the blood to clot within 30 min, then centrifuged for 10 min, and then the serum was stored at 2–8°C. Because of the diurnal difference in cortisol levels, blood samples were drawn at a specific time of day (6–10 am). We measured CoQ10 using an ELISA (Sino Gene Clon Biotech Co., Ltd) with cut-off ranges of 1.56 ng/ml, 1.56 ng/ml, and 50 ng/ml (19).

### Statistical Analysis

We used SPSS version 22(SPSS Inc., Chicago, IL, USA) to analyze the study data. Qualitative data were expressed as number (%), while quantitative data were expressed as mean ± SD and range. Linear regression analysis was performed to assess the predictors of burnout, strain, and engagement as outcomes to technostress creators. Then, we finally analyzed the risk factors associated with technostress through binary logistic regression between low + moderate technostress vs. high technostress either among students or the staff. Multiple comparisons were tested using Holm-Bonferroni Sequential Correction: An EXCEL Calculator © Justin Gaetano, 2013, and *p-*values are statistically significant after this correction.

### Compliance With Ethical Standards

This study was commenced after obtaining ethical approval from the Research Ethical Committee (REC) of Menoufia Faculty of Medicine, Menoufia University (ID: 06/2021FAM). The REC approved the holistic approach including the questionnaire and withdrawing blood samples, for blood cortisol and co-enzyme Q10 levels, under aseptic sterile conditions which were considered a minimally invasive low risk intervention. Moreover, informed consent was taken from every participant after being informed of all aspects of the study. The data, including responses to the questionnaire and the laboratory workup, were handled anonymously to maintain the confidentiality of the participants.

## Results

We recruited a total of 3,582 participants, 1,056 staff members aged 36.1 ± 9.7 years, and 2,526 students aged 20.2 ± 1.3 years. Among the staff members, 69.7% were females and 51.1% were urban residents; they spent a mean of 35.5 ± 24.4 h/week using ICTs. Among the students, 80.5% were females and 61.8% were urban residents. The students spent a mean of 40.1 ± 27.8 h/week with ICTs (Table 1).

**Table 1:** Characteristics of the studied staff and students.

**Staff (No = 1,056)**	**Mean ±SD**	**Range**
Age (y)	36.1 ± 9.7	26–65
Experience years	11.3 ± 8.2	0–35
Technical hours/ week	35.5 ± 24.4	0–84
	no	%
Gender		
Male	320	30.3
Female	736	69.7
Department		
Academic	264	25.0
Clinical	792	75.0
Residence		
Rural	516	48.9
Urban	540	51.1
Program training	711	67.3
Smart devices	993	94.0
Good network connection		
Yes	679	64.3
Sometimes	377	35.7
**Students (No** **=** **2526)**	**Mean** **±SD**	**Range**
Age (y)	20.2 ± 1.3	18–24
Technical hours/ week	40.1 ± 27.8	2–140
	no	%
Gender		
Male	492	19.5
Female	2,043	80.5
Education stage		
Pre-clinical	1,860	73.6
Clinical	666	26.4
Residence		
Rural	1,562	61.8
Urban	964	38.2
Smart devices	2,481	98.2
Good network connection		
Yes	1,691	66.9
Sometimes	835	33.1

The mean total technostress score was 3.44 ± 0.48 vs. 2.91 ± 0.50 among medical students. For the technical characteristics, the staff gave high scores to IT presenteeism (3.94 ± 0.95) and pace of IT change (3.99 ± 0.82), whereas they gave task interdependence a high score (3.85 ± 0.760) for the job characteristics vs. 3.95 ± 0.80, 3.74 ± 0.81, and 3.21 ± 1.01, respectively, among students (Table 2).

**Table 2:** Technostress creators, job and technology characteristics among the studied staff and students.

		**Staff (No** **=** **1,056)**		**Students (No** **=** **2,526)**	
		**Mean ±SD**	**Range**	**Mean ±SD**	**Range**
Techno–stress creators	•Techno–overload	3.74 ± 0.70	2–5	3.98 ± 0.83	1–5
	•Techno–invasion	3.91 ± 0.93	1–5	3.93 ± 0.87	1–5
	•Techno–complexity	3.47 ± 0.87	1.4–5	3.45 ± 0.85	1–5
	•Techno–insecurity	3.10 ± 0.69	1.5–5.0	2.56 ± 0.67	0.8–4.4
	•Techno–uncertainty	3.0 ± 0.47	2–5	0.64 ± 0.16	0.2–1.10
	•Total	3.44 ± 0.48	2.16–4.60	2.91 ± 0.50	0.95–4.0
Technology characteristics	•IT complexity	2.41 ± 0.99	1–5	2.62 ± 0.97	1–5
	•IT presenteeism	3.94 ± 0.95	1–5	3.95 ± 0.80	1–5
	•Pace of IT change	3.99 ± 0.82	1–5	3.74 ± 0.81	1–5
Job characteristics	•Job autonomy	3.50 ± 0.84	1–5	3.21 ± 1.01	1–5
	•Task interdependence	3.85 ± 0.76	1.33–5.0	3.95 ± 0.75	1–5

**Figure 2:**
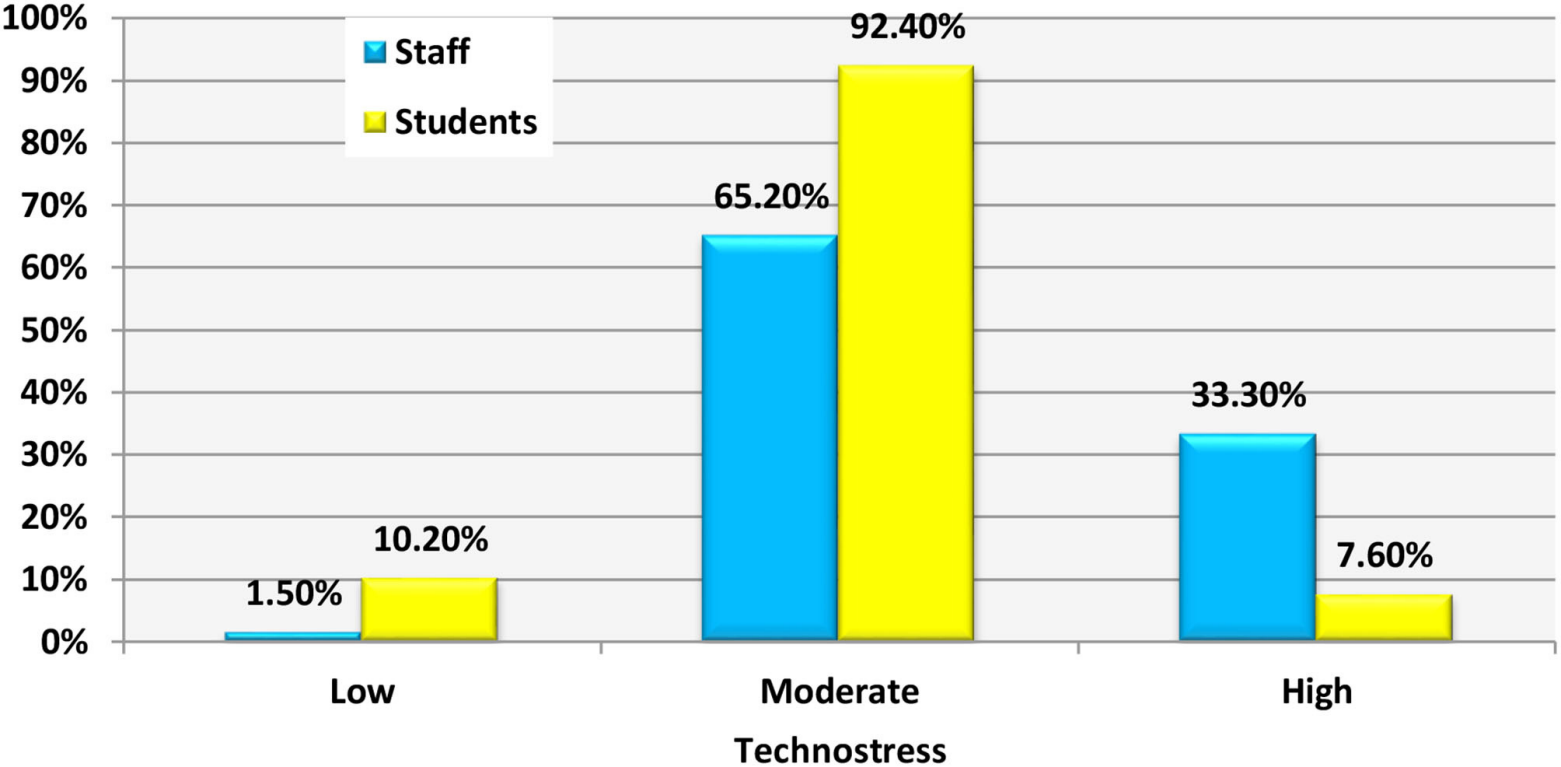
Distribution of the studied groups regarding the prevalence of technostress.

The technostress score was distributed as 33.3% high, 65.2% moderate, and 1.5% low technostress among the medical staff while among the students, it was high in 7.6% of them, 92.4% moderate, and 10.2% low (Figure 2).

### Among the Medical Staff

Linear regression analysis was conducted to assess the predictors to burnout, strain, and engagement as outcomes to technostress creators and job and technology characteristics:

#### Burnout

**Figure 3:**
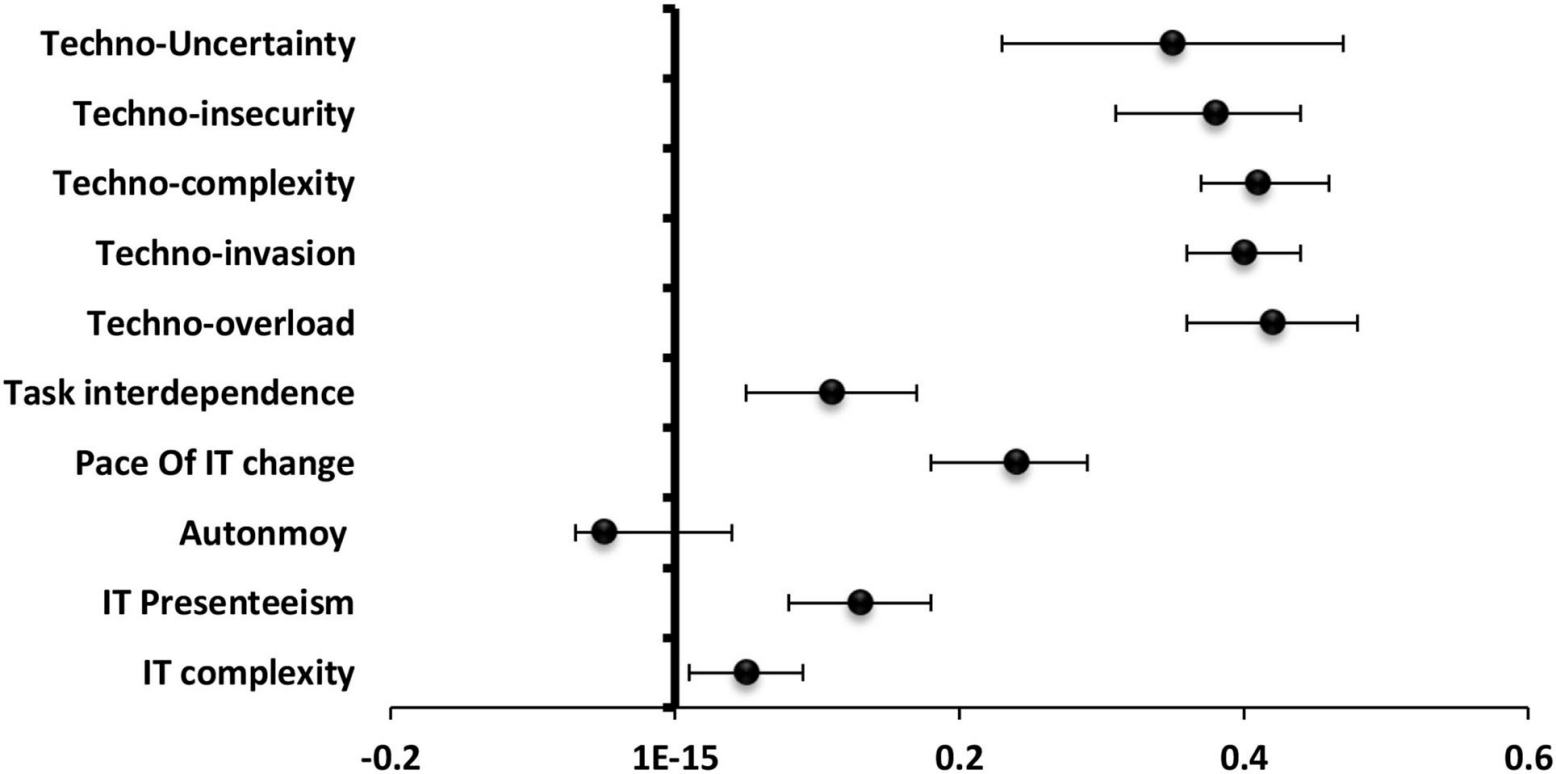
Values of β and CI 95% for linear regression analysis of technostress items and job and technology characteristics as a predictor to burnout among staff.

The most significantly positive predictor to burnout among technostress creators was techno-overload (β = 0.42) followed by techno-invasion (β = 0.40), techno-complexity (β = 0.41), techno-insecurity (β = 0.38), and techno-uncertainty (β = 0.35). For **job and technical characteristics, the** pace of IT change (β = 0.24) was the most significantly positive predictor to burnout followed by IT presenteeism (β = 0.13) and task interdependence (β = 0.11) (Figure 3).

#### Strain

**Figure 4:**
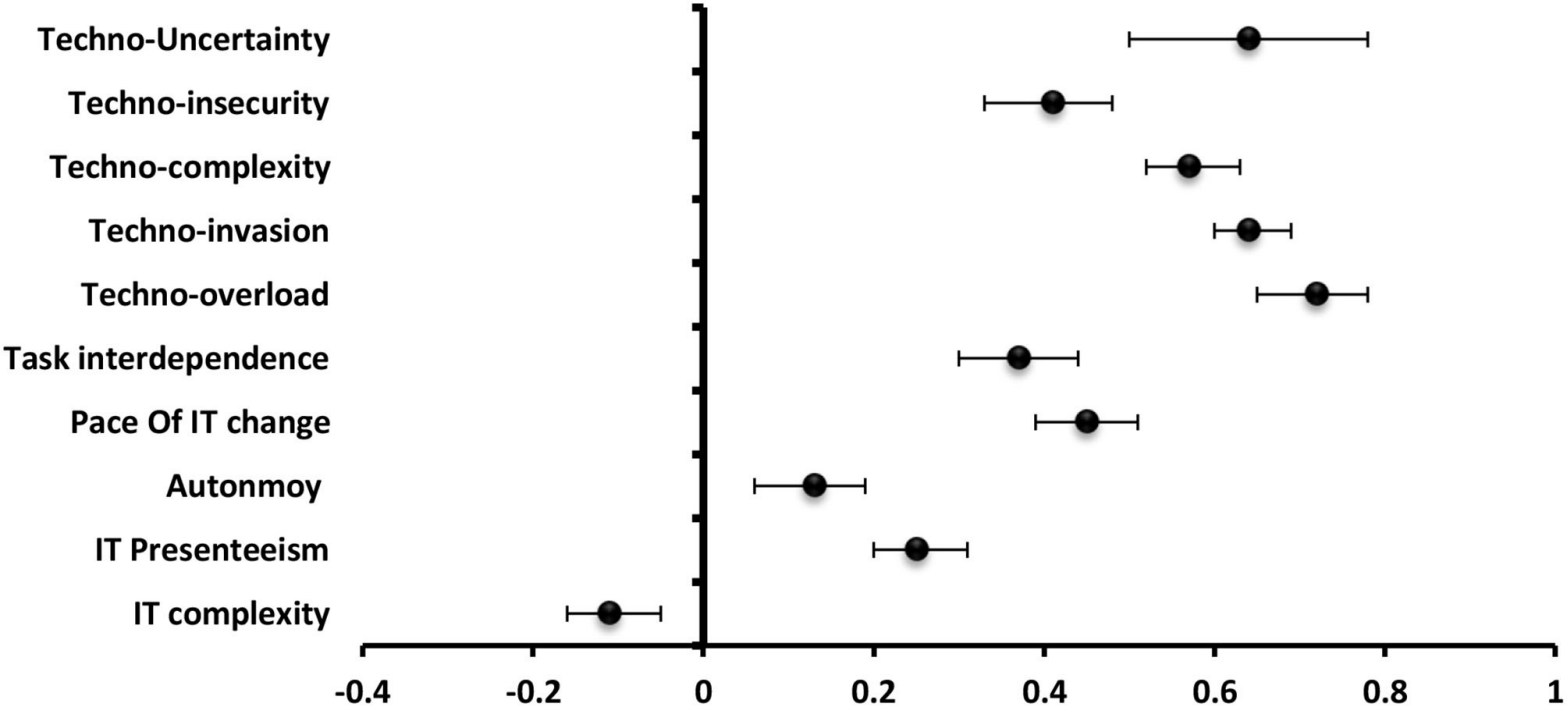
Values of β and CI 95% for linear regression analysis of techno-stress items and job and technology characteristics as a predictor to strain among staff.

**The** most significantly positive predictor to strain among **technostress creators** was techno-overload (β = 0.72), techno-invasion (β = 0.64), techno-uncertainty (β = 0.64), techno-complexity (β = 0.57), and techno-insecurity (β = 0.41). For **job and technology characteristics, the** pace of IT change (β = 0.45) was the most significantly positive predictor followed by task interdependence (β = 0.37), IT presenteeism (β = 0.25), and job autonomy (β = 0.13) while IT complexity (β = −0.11) served as a significantly negative predictor to strain (Figure 4).

#### Engagement

**Figure 5:**
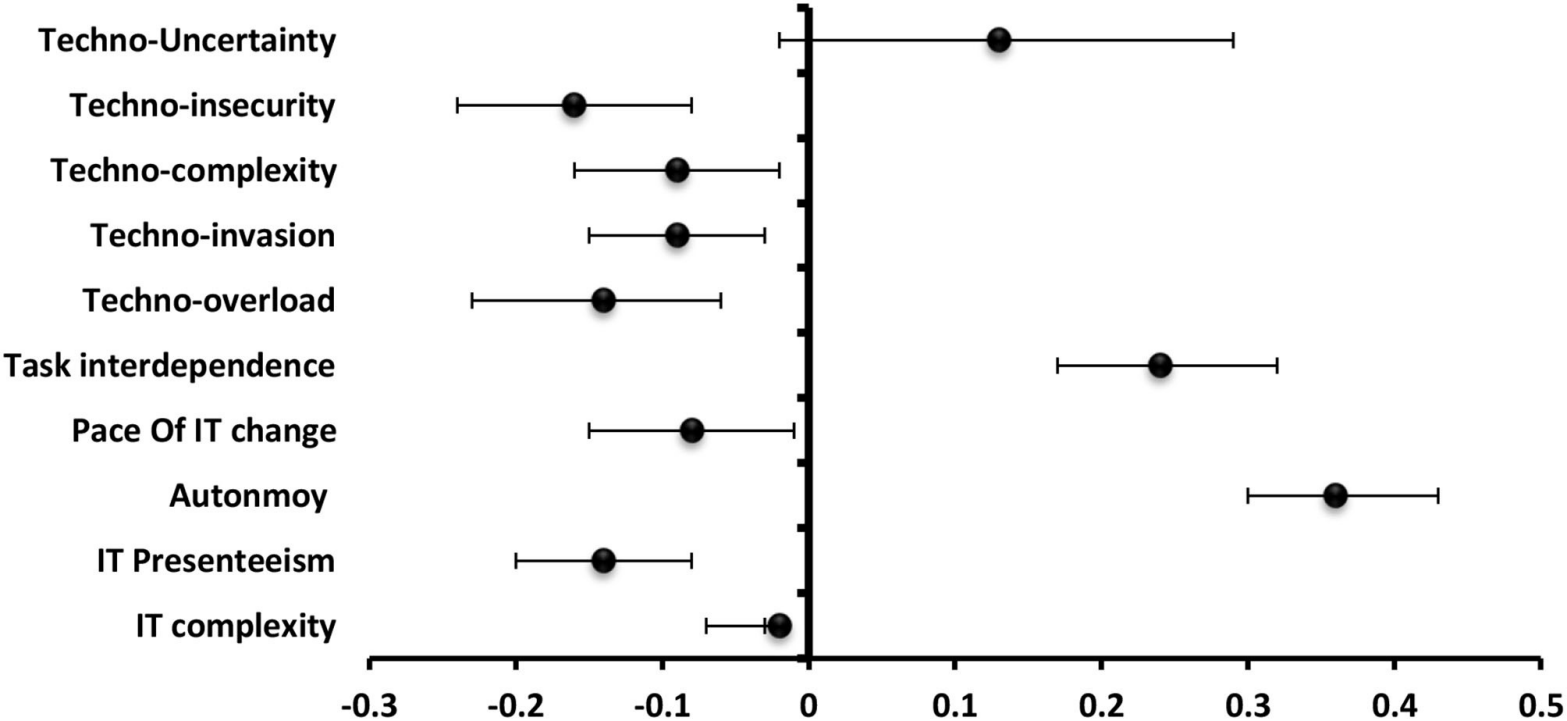
Values of β and CI 95% for linear regression analysis of techno-stress items and job and technology characteristics as a predictor to engagement among staff.

**The** most significantly negative predictor to engagement among **technostress creators** was techno-insecurity (β = −0.16) followed by techno-overload (β = −0.14), techno-invasion (β = −0.09) and techno-complexity (β = −0.09). For **job and technology characteristics**, job autonomy (β = 0.36), and task interdependence (β = 0.24) were significantly positive predictors while IT presenteeism (β = −0.14) and pace of IT change (β = −0.08) were significantly negative predictors (Figure 5).

### Among the Medical Students

Linear regression analysis was conducted to assess the predictors to burnout, strain, and engagement as outcomes to technostress creators and job and technology characteristics:

#### Burnout

**Figure 6:**
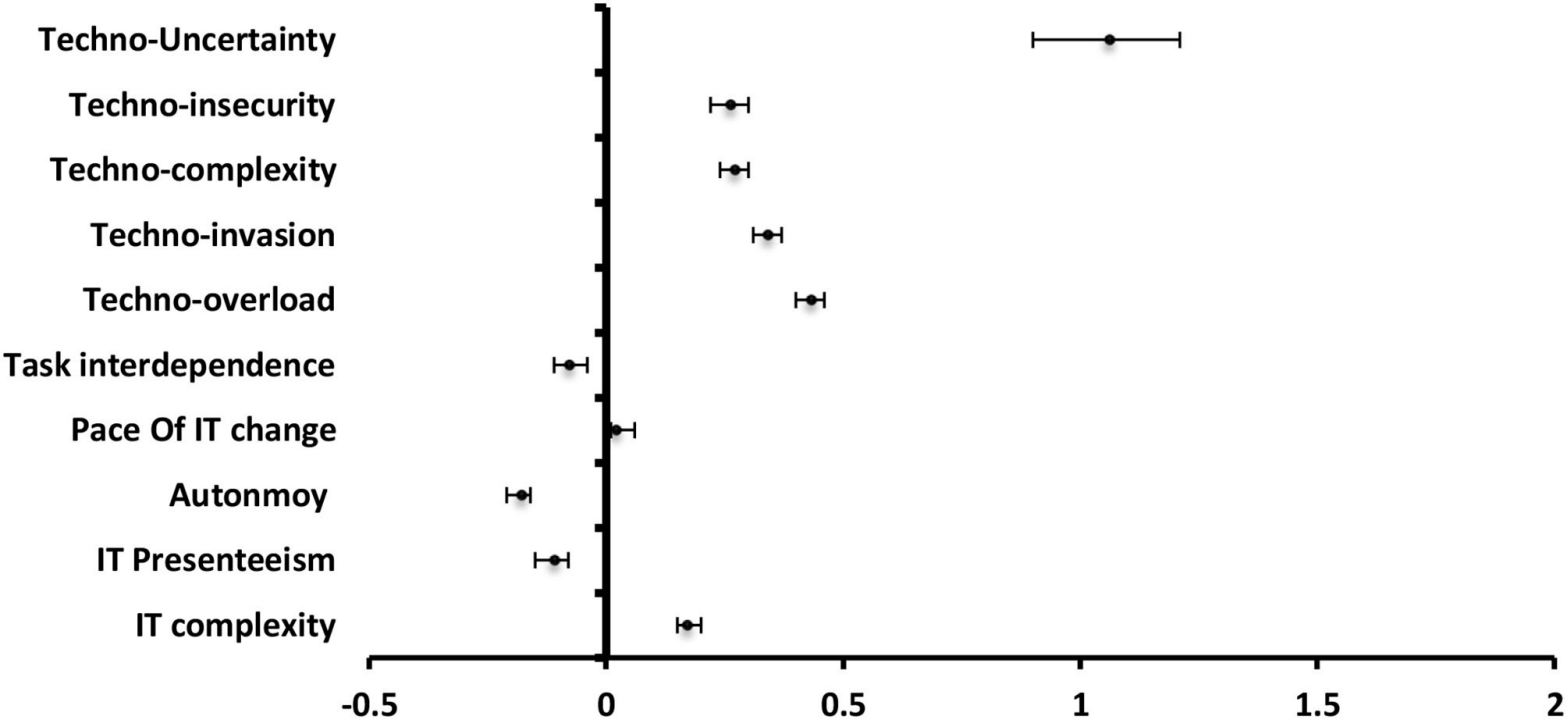
Values of β and CI 95% for linear regression analysis of techno-stress items and job and technology characteristics as a predictor to burnout among students.

**The** most significantly positive predictor to burnout among **technostress creators** was techno-uncertainty (β = 1.06) followed by techno-overload (β = 0.43,), techno-invasion (β = 0.34), techno-complexity (β = 0.27), and techno-insecurity (β = 0.26). For **job and technology characteristics**, IT complexity (β = 0.17) served as a significantly positive predictor to burnout while, job autonomy (β = −0.18), IT presenteeism (β = −0.11), and task interdependence (β = −0.08) were significantly negative predictors in order (Figure 6).

#### Strain

**Figure 7:**
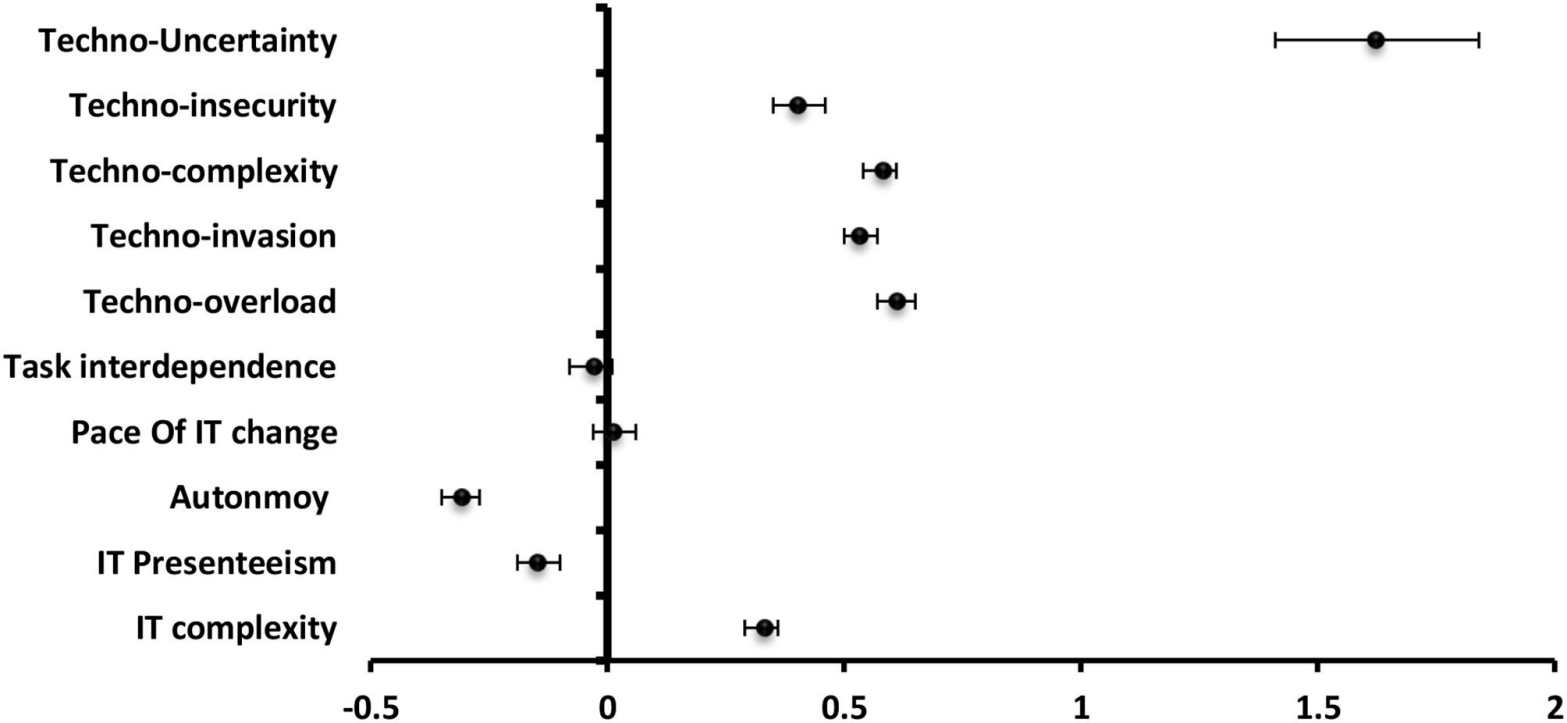
Values of β and CI 95% for linear regression analysis of techno-stress items and job and technology characteristics as a predictor to strain among students.

**The** most significantly positive predictor to strain among **technostress creators** was techno-uncertainty (β = 1.62) followed by techno-overload (β = 0.61), techno-complexity (β = 0.58), techno-invasion (β = 0.53), and techno-insecurity (β = 0.40). For **job and technology characteristics**, IT complexity (β = 0.33) served as a significantly positive predictor to strain while job autonomy (β = −0.31) was the most significantly negative predictor (Figure 7).

#### Engagement

**Figure 8:**
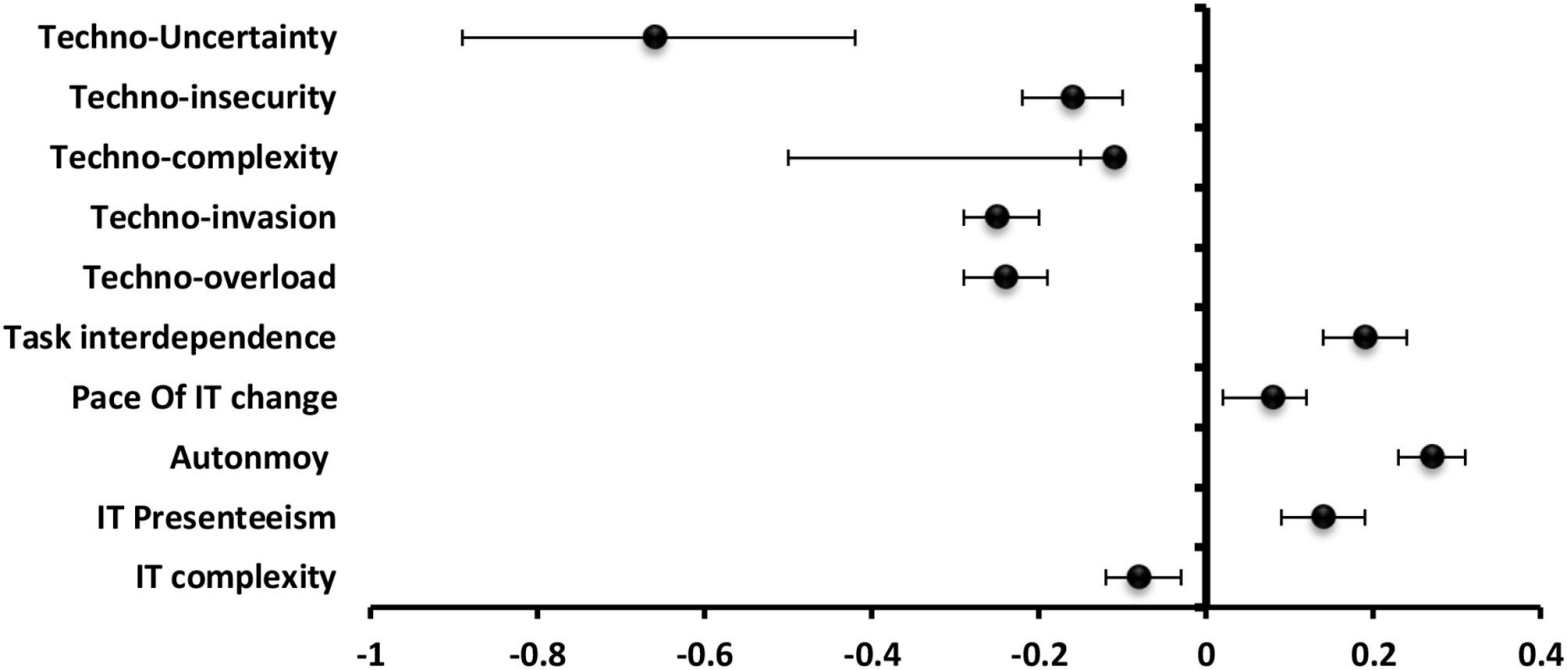
Values of β and CI 95% for linear regression analysis of techno-stress items and job and technology characteristics as a predictor to engagement among students.

**The** most significantly positive predictor to strain among **technostress creators** was techno-uncertainty (β = −0.66) followed by techno-invasion (β = −0.25), techno-overload (β = −0.24), techno-insecurity (β = −0.16). For **job and technology characteristics**, job autonomy (β = 0.27), and IT presenteeism (β = 0.14) was the most significantly positive predictor while techno-complexity (β = −0.11) was the most significantly negative one to engagement (Figure 8).

Linear regression analysis was conducted to assess the predictors to burnout, strain, engagement, and biomarkers (Cortisol and CoQ10) as outcomes to technostress total score.

**Figure 9:**
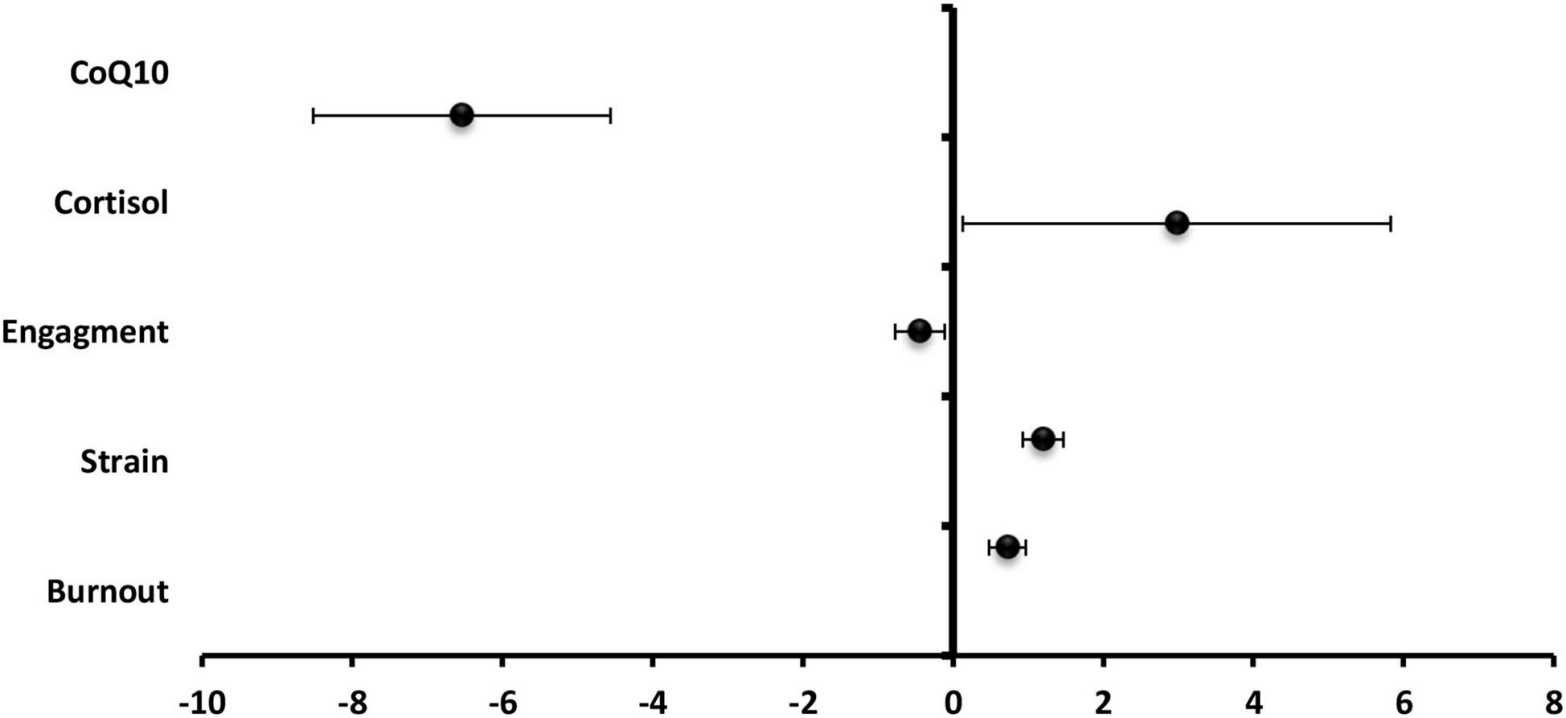
Values of β and CI 95% for linear regression analysis of total techno-stress score as a predictor to biomarkers, burnout, strain, and engagement among staff.

**Figure 10:**
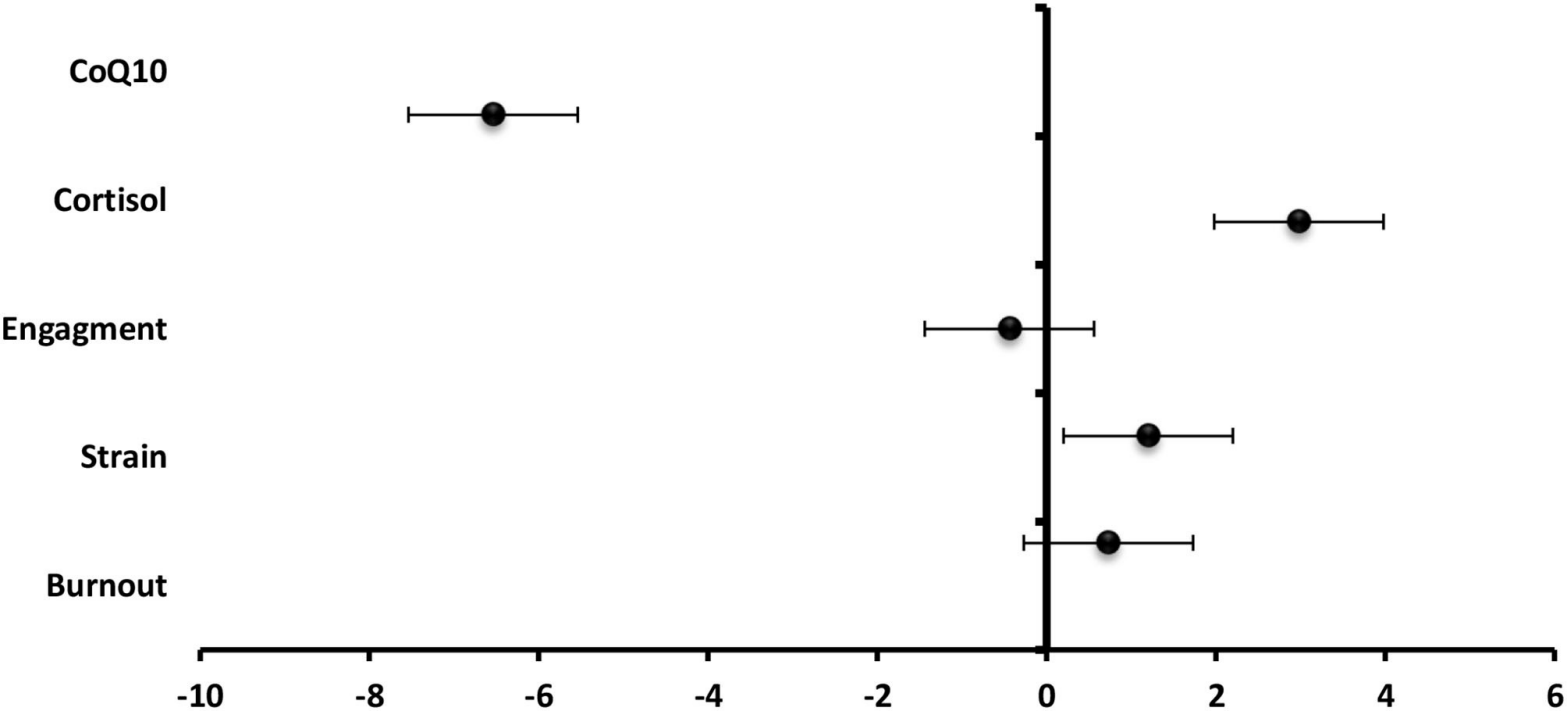
Values of β and CI 95% for linear regression analysis of total techno-stress score as a predictor to biomarkers, burnout, strain, and engagement among students.

**Figure 11:**
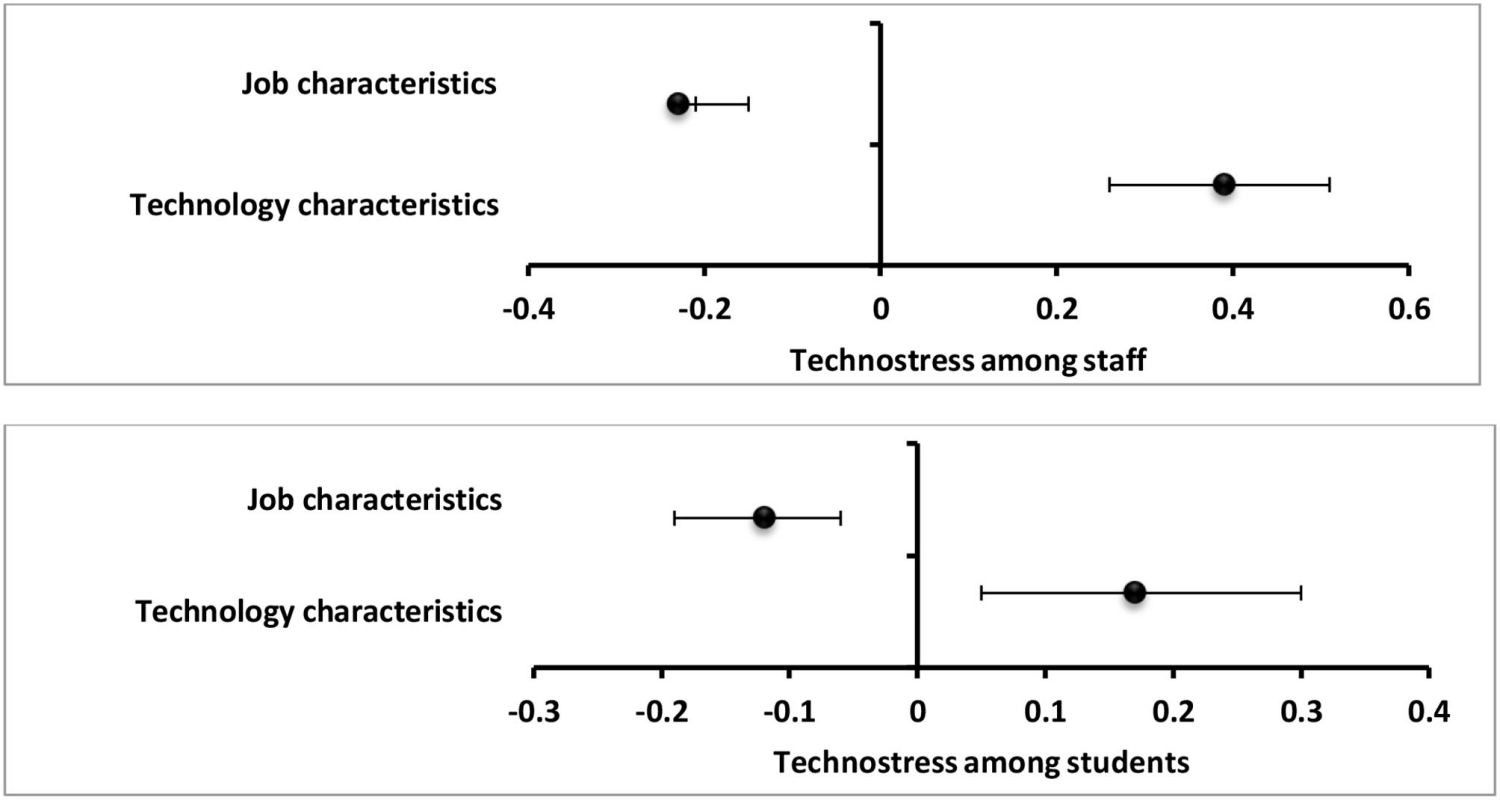
Values of β and CI 95% for linear regression analysis of job characteristics and technology characteristics as predictors to total techno-stress score among staff and students.

- Among staff members, total technostress score significantly positive predictor to predicted cortisol level (β = 2.98), strain (β =1.20), and burnout (β = 0.73) and significantly negative predictor to engagement (β = −0.44) and CoQ10 (β = −6.54) (Figure 9).
- Among the students, total technostress scores significantly positive predictor to cortisol level (β = 6.64), strain (β = 1.25), and burnout (Figure 10).
- Technology characteristics were significantly positive predictor to technostress among staff members and students (β = 0.39 and β = 0.17), respectively, while job characteristics were significantly negative technostress predictors (β = −0.23 and β = −0.12,) respectively (Figure 11).
- Binary logistic regression analysis was conducted to assess the risk factors associated with technostress and it revealed that rural residence, increasing technical hours/week, IT complexity, the pace of change, job autonomy, and task interdependence were significant risk factors for technostress among the studied staff members (*p* < 0.001) whereas female gender, rural residence, low-educational stage, increasing technical h/week, the pace of change, and job autonomy were significant risk factors among the studied undergraduate medical students (Table 3).

**Table 3:** Risk factors for technostress among staff and students.

**Staff**	**β**	***P* value**	**95%CI**	
			**Lower bound**	**Upper bound**
Residence (rural)	−0.133	<0.001^*^	−0.184	−0.082
Technical hours/week	0.006	<0.001^*^	0.005	0.007
IT complexity	0.075	<0.001^*^	0.051	0.100
Pace change	0.261	<0.001^*^	0.223	0.300
Job autonomy	−0.058	<0.001^*^	−0.088	−0.027
Task interdependence	−0.141	<0.001^*^	−0.179	−0.103
Presenteeism	−0.037	0.012	−0.066	−0.008
Experience years	−0.011	0.020	−0.020	−0.002
Gender (female)	0.053	0.035	0.004	0.103
**Students**				
Gender (female)	0.136	<0.001^*^	0.093	0.178
Residence (rural)	0.102	<0.001^*^	0.068	0.136
Grade (low grade)	0.058	<0.001^*^	0.027	0.090
Educational stage	−0.253	<0.001^*^	−0.311	−0.195
Technical hours/week	0.002	<0.001^*^	0.002	0.003
IT complexity	0.197	<0.001^*^	0.179	0.214
Pace change	0.096	<0.001^*^	0.075	0.117
Job autonomy	−0.140	<0.001^*^	−0.158	−0.121
Task interdependence	−0.047	<0.001^*^	−0.071	−0.023
Age	0.041	0.001^*^	0.018	0.065

* *Significant*.

## Discussion

One of the consequences of the global COVID-19 pandemic has been the dramatic changes to working environments globally. In 2020, for many employees, remote work using ICTs became a need rather than a luxury. In the previous research, healthcare workers (staff and students) reported high stress owing to this shift (7, 20). Technostress has been described by scientists as the dark side of technology use (21). This study examined technostress among healthcare faculty members at the Egyptian University and undergraduate medical students in different educational contexts.

The results revealed the prevalence of high-to-moderate technostress among medical staff members and students. These results were consistent with findings of another Egyptian study carried out among University staff members (20).

Healthcare professionals have reported that the technostress is significantly higher than that found among workers in other occupations, possibly due to higher job requirements, including teaching, research, and clinical practice (22). Outside of both Egypt and health care, about 80% of librarians at different African universities experience technostress (23, 24).

Differences in the prevalence and levels of technostress between this study and previous research can be attributed to differences in contexts. Librarians, especially now in sophisticated libraries in the 21st century, are more involved in computer technologies than in other fields.

This study, we found that staff members with less ICT experience but more technical h / week were more at risk for technicians and that being a student and living in a rural area were other technostress-related risk factors. These results are consistent with the vast majority of previous findings for more technostress among faculty members who were older, had more teaching experience, and were females living in rural areas (20). Conversely, other researchers found no significant differences in technostress incidence between males and females (25, 26). The inconsistent findings of this study could be the result of the different learning contexts and cultural concepts incorporated into other studies.

This study revealed that technology characteristics are remarkably positive predictors of technostress. The positive association between technology complexity and presenteeism and pace of change on the one hand and technostress on the other side agrees with many studies (26, 27).

Fortunately, Shu et al. considered technology complexity to be a modifiable risk factor for technostress and recommended updating computer self-efficacy and faculty competencies to overcome the resulting technostress (6).

The striking negative association between job characteristics (autonomy and task interdependence) and technostress in this study is noteworthy. Job autonomy describes the situation in which workers have the power to make decisions regarding their occupational tasks. In agreement with this study, autonomy has been reported to be negatively associated with technology (1).

During the COVID-19 pandemic, both staff and students are becoming more autonomous. Working from home emphasized job autonomy and required most staff members to coordinate, supervise, manage their tasks themselves, and make decisions more autonomously than ever before (5).

Mali Wong et al. not only the amount of interdependence that influences stress, but the clarity of interrelated tasks, worker roles, and the direction of interdependence, both extrinsic and intrinsic (28). Integrating alternative communication mechanisms to overcome social isolation and deficient collaborative work is expected to be promising.

Task interdependence is an integral part of today's organizations, and it describes the extent to which work teams interact to achieve a specific task (28). Turetken et al. and Tarafdar supported our study for the negative impact of task interdependence on the technostress and productivity during virtual work. They attributed this to the lack of interaction between the employee who works from home, which disturbs coordination, physical interaction, and subsequent achievement (29, 30). In Wong et al., it is not just the amount of interdependence that influences stress, but the clarity of interdependence tasks, the roles of workers, and the direction of interdependence, both external and internal (28). Integrating alternative communication mechanisms to overcome social isolation and deficient collaborative work is expected to be promising (28).

Burnout is defined as an inadequate response resulting from chronic work stress. It involves three components; depersonalization, emotional exhaustion, and low achievement (31).

Burnout was among the outcomes evaluated in this study. It was determined that techno-overload was the strongest contributor to fatigue among medical school faculty, while techno-uncertainty was the strongest contributor among medical students. With this study, we were the first to identify techno-overload as a contributor to technostress among faculty and the techno-uncertainty among medical students. Techno-overload is defined as the high demand for work that requires work faster and longer than usual to meet work obligations (26), and some researchers found that work overload was the primary contributor to technostress (21). Burnout occurs when the demands of a job exceed an individual's ability to adapt (32); it is a psychological behavioral disorder that appears by individuals under constant stress. Researchers have established a precise association between techno-overload (workload and work pressure) and burnout (23, 33). The results were more pronounced in medical fields because of the central nature of medical schools and the lower scope of innovation and creativity in the medical sciences compared to other fields. These factors contribute to problems such as burnout (33).

The techno-invasion scores in this study, 3.91 ± 0.93 for staff and 3.93 ± 0.87 for students, were lower than the averages reported in a previous Egyptian study in a similar educational setting (6.61 ± 2.76) (20). The inconsistency can be attributed to the different questionnaires used in evaluating technostress and the time periods in which the study in question was conducted. Gabr et al. (16) conducted their study in December 2020, before we conducted our study when learning was completely virtual due to the lockdown. Conversely, we conducted this study between January and May 2021, when learning was mainly mixed. This distinction could also explain the significant discrepancy between participants' ratings of ICT complexity in this study, which were 3.47 ± 0.87 for staff and 3.45 ± 0.85 for students, compared with the mean of 12.47 ± 4.20 reported by Gabr et al. (16). Participants in the latter study were fully engaged with ICTs and the online learning environment, whereas participants in this study reported more ICT training and modern device use.

Working strain is another important finding that this study addressed in detail. The strain is well-described based on the stress–strain model. When an individual suffers from certain psychological stresses (related to the work environment) that exceed his resources and capabilities, he suffers from a negative emotional feeling (strain) (34).

Techno-invasion, yet another of the dimensions of technostress, appeared in this study and it was associated with more burnout, strain, and less work engagement. The researchers describe technical invasion as the loss of privacy in one's personal life due to information and communication technology. When technology makes workers reachable at any time, with no boundaries between work and personal time, individuals report considerable work–family conflicts (20).

Similar to this study, Molyneux et al. (2020) accurately described the associations between techno-overload, techno-invasion, and stress, and found that conflict between family and work has significant direct correlations with technostress subscales. The authors blamed the work-at-home environment and at the same time expressed that universities are insisting on employees to continue working virtually, at least part-time, even after the lockdown is lifted, unsuitable for work (35). In contrast to this study, some research has reported positive effects of prolonged virtual work of more than a year on family and work conflict compared to actual work of <1 year (4). We consider it worth noting that most of the medical students in this study (1,562 of 2,526) and approximately half of the faculty members were living in the rural areas and might have been using ICTs for the first time.

Techno-uncertainty is another contributor to technostress, and it was identified as one of the strongest contributors to burnout and strain among the participants in this study. Techno-uncertainty refers to discomfort concerning the use of ICTs at work (33), and researchers identified its influence on worker strain (14) and fatigue (21). Apprehension, anxiety, and agitation are common behavioral strains attributable to technostress, and one marker of techno-uncertainty, in particular, is fear of losing information from clicking incorrect keys or generally making mistakes (36).

Engagement in work is a known term that refers to which extent the employees are committed to their work. However, the literature defined the engagement of students in a different way. It was described by the ability of students to behave, recognize, and feel in the expected way during the learning process (37).

Furthermore, we found that techno-insecurity positively correlated with burnout and strain and negatively with work engagement. Techno-insecurity is defined as the constant fear of losing one's job or being replaced with an employee with greater ICT capabilities (33); researchers associated it with emotional exhaustion, burnout, and strain; employees require specific coping mechanisms to reduce the harmful emotional stress owing to techno-insecurity (38).

Consistent with the findings of this study, previous researchers found negative correlations between techno-overload, techno-invasion, and techno-uncertainty on one side and work engagement on the other (33). They revealed that better faculty work performance was associated with these three technostress dimensions in particular, and suggested three strategies to counteract technostress: techno-support anticipation, facilitation of ICT literacy, and involvement (33). However, contrary to this study, scholars found positive correlations between job satisfaction and, in turn, engagement and virtual work and telecommunication; notably, though, the correlation was weak (r = 0.09, 95% CI: 0.07−0.11) (4). Previous researchers found a curvilinear relationship between workload and job satisfaction and subsequent engagement; working for more than 15.1 h/week was associated with a significant decline in job satisfaction and engagement compared with working fewer hours (39, 40). The fact that the workloads of the participants in this study were 35.5 ± 24.4 h/week for the faculty members and 40.1 ± 27.8 for the students could explain the discrepancies between the findings of this study and the previous ones (39, 40).

This study indicated that among the staff members, presenteeism was a positive predictor of burnout, strain, and a negative predictor of work engagement. Presenteeism describes the phenomenon of appearing at work without being productive, and burned-out employees who are indecisive show more presenteeism, absence, and turnover. Therefore, the researchers described presenteeism as a risk factor for burnout and highlighted its negative influence on work engagement and achievement (41). Researchers demonstrated that burnout can weaken “the gain cycle of daily job resources, daily work engagement, and daily job crafting” (41). Consistent with the findings here, other researchers found that technostress with resultant burnout negatively affected worker productivity (42), efficiency (43), job satisfaction, and ongoing commitment (44).

The findings of this study clearly contrast with other previous findings. For instance, virtual work was more efficient because it enabled completing tasks from anywhere and consequently lead to increased efficiency, employee satisfaction, and balance between work and family life (45). Moreover, researchers associated virtual work with better work performance and lower work-role stress (4) and found that ICTs can expedite task completion, thereby improving quality-of-life (21). Another researcher argued against any association between technostress and students' academic performance (46). The discrepancies between our findings and these contrasting results might be attributable to the variations in the working contexts. The participants in the aforementioned studies mentioned were business users of ICTs, in contrast with our medical school faculty members and undergraduate students.

Barring the behavioral and psychological impacts of technostress, we here shed light on the influence of technostress on human biological systems. This study depicts that the total technostress score predicted blood cortisol level in both staff and students and found significant positive correlations with burnout, strain, and cortisol level. These results were consistent with previous findings of higher blood cortisol among study participants with higher technostress subscale scores, specifically, techno-overload and IT complexity (20). Moreover, Adam (2006) connected higher diurnal cortisol level and secretion in response to a stressor with increased self-reported pressure and negative mood (47).

Cortisol secretion is mediated by the hypothalamus-hypophyseal tract where the thalamus and the frontal cortex integrate sensory stimuli in response to different technostress creators. The brain sends this information to the limbic system, which mediates the emotional responses, and the hypothalamus releases a corticotrophin-releasing hormone (CRH). The CRH stimulates the pituitary gland to release an adrenocorticotropic hormone into the blood; subsequently, the adrenals secrete cortisol, which mediates the behavioral responses (48). Stress-induced cortisol secretions enable the human body to adapt perception, memory, cognition, and behavior to stressors (49). However, in the long run, cortisol precipitates burnout, depression, anxiety, hypertension, atherosclerosis, and immunological disorders (50–52).

This study, total technostress score was a significant predictor ofCoQ10 among studied staff, and researchers established a negative correlation betweenCoQ10 and certain working conditions. Co-enzyme Q10 is a vitamin-like antioxidant that is believed to exert protective effects on different body systems, notably, the cardiovascular system; it has a crucial role in producing cellular energy (53). The findings of negative correlation between technostress and CoQ10 are consistent with the recent study findings of a significant correlation between low CoQ10 and excessive working and burnout from a study on healthcare workers in Egypt (19).

Despite the ambiguity in study findings related to the exact role of CoQ10, researchers established a role of low CoQ10 in the path physiology of depression (54, 55). Consistent with this study, previous studies revealed that overwork was associated with burnout and that burnout significantly predicted the inflammatory cytokines TNF-a, IL6, and CoQ10 (23, 51, 52). Through their effects on the central nervous system, these cytokines precipitate behavioral manifestations, such as fatigue, diminished appetite, and inhibited libido (56).

### Strength and Limitations

This study evaluates the prevalence and creators of technostress among medical staff and students as well as addresses the behavioral and biological consequences (fatigue, stress, engagement, cortisol, and CoQ10 levels) helping to better understand the underlying mechanisms and sequencing of technostress. Involving participants of different ranks from different universities increases the reliability of this study and allows generalization of the results obtained. Furthermore, as recall bias might be there, we attempted to offset this by looking at the assessment of cortisol and coenzyme Q10 levels in some of the participants. The main limitation of this study is limited to University healthcare workers (colleges and medical students) except healthcare workers outside the University. However, future research should be directed to other business sectors in healthcare, investigating potential adverse effects of technology and offering different adaptation strategies to deal with technology resulting from ICT due to the current pandemic. Another recommendation is to consider personality traits while suggesting coping strategies to overcome the technique.

## Conclusion

Faculty and students at the Egyptian Colleges of Medicine and Health Care report that they encounter medium-to-high tech related to their use of ICT. In this study, higher stress was associated with extreme burnout, strain, and cortisol level on the one hand and lower engagement in work and CoQ10 on the other. The findings highlighted that it may be beneficial for medical school administrators to adopt programs to facilitate staff and students in the use of ICTs during the COVID-19 period of virtual work; such programs may include psychological support for individuals who are struggling. These facilitation programs should include training in creating good networks, use of smart devices, and IT support teams for staff and students as cornerstones to overcoming technology. Furthermore, healthcare and medical professionals must devote time and space to virtual work and not blur the boundaries between work and home, and policymakers must suggest different strategies for adapting to technostress.

## Data Availability Statement

The dataset analyzed during the current study are available from authors upon justified request.

## Ethics Statement

The current study was carried out following the Declaration of Helsinki. Data collection was commenced after obtaining approval from Research Ethical Committee REC of Menoufia Faculty of Medicine, Menoufia University (ID: 06/2021FAM). An informed consent were taken in which each participant has been informed of all aspects of the study and have the right to give up as he/she wanted. The data were handled anonymously to maintain the confidentiality of the participants. The patients/participants provided their written informed consent to participate in this study.

## Author Contributions

ZAK conceptualized the study, analyzed and interpreted the data, wrote the manuscript draft, proofread it and approved the final manuscript. AFS collected the data, wrote discussion, and revised methods and approved the final manuscript. AMB formulate the questionnaire and wrote the methods. AYS, NNH, and HMA conceptualized the study and collected the data, and approved the final manuscript. ASE and KAA conceptualized the idea and collected the data. SRA and NHH collected the data, wrote introduction, and approved the final manuscript. SMA collected the data, wrote discussion, and revised methods and approved the final manuscript.

## Conflict of Interest

The authors declare that the research was conducted in the absence of any commercial or financial relationships that could be construed as a potential conflict of interest.

## Publisher's Note

All claims expressed in this article are solely those of the authors and do not necessarily represent those of their affiliated organizations, or those of the publisher, the editors and the reviewers. Any product that may be evaluated in this article, or claim that may be made by its manufacturer, is not guaranteed or endorsed by the publisher.
